# Efficacy and safety of tisagenlecleucel in adult Japanese patients with relapsed or refractory follicular lymphoma: results from the phase 2 ELARA trial

**DOI:** 10.1007/s12185-022-03481-y

**Published:** 2022-11-21

**Authors:** Noriko Fukuhara, Koji Kato, Hideki Goto, Tajima Takeshi, Mayu Kawaguchi, Kota Tokushige, Koichi Akashi, Takanori Teshima, Hideo Harigae, Stephen J. Schuster, Catherine Thieblemont, Martin Dreyling, Nathan Fowler

**Affiliations:** 1 Department of Hematology, Tohoku University Hospital, 1-1 Seiryo-cho, Sendai, 980-8574 Japan; 2 Department of Medicine and Biosystemic Science, Kyushu University Graduate School of Medical Sciences, Fukuoka, Japan; 3 Department of Hematology, Faculty of Medicine and Graduate School of Medicine, Hokkaido University, Sapporo, Japan; 4 Novartis Pharma K.K, Toranomon Minato-ku, Tokyo, Japan; 5 Lymphoma Program, University of Pennsylvania, Philadelphia, PA USA; 6 APHP, Hôpital Saint-Louis-Université de Paris, Paris, France; 7 Department of Internal Medicine III, LMU Hospital, Munich, Germany; 8 The University of Texas MD Anderson Cancer Center, Houston, TX USA; 9 BostonGene, Waltham, MA USA

**Keywords:** CAR-T, ELARA, Relapsed or refractory follicular lymphoma, Tisagenlecleucel

## Abstract

**Background:**

Tisagenlecleucel yielded a high durable response rate in patients with relapsed/refractory (r/r) follicular lymphoma (FL) in the global phase 2 ELARA trial. Here, we report the efficacy, safety, and cellular kinetics of tisagenlecleucel in a subgroup of Japanese patients with r/r FL from ELARA.

**Methods:**

ELARA (NCT03568461) is a global single-arm trial of tisagenlecleucel in patients with r/r FL who received ≥ 2 prior lines of therapy. The primary endpoint was the complete response rate (CRR), and the secondary endpoints were the overall response rate, duration of response, progression-free survival, overall survival, safety, and cellular kinetics.

**Results:**

As of March 29, 2021, nine Japanese patients were enrolled and received tisagenlecleucel with a median follow-up of 13.6 months (range, 10.5‒19.3). Per independent review committee, CRR was 100% (95% CI 63.1‒100). Within 8 weeks of infusion, cytokine release syndrome (CRS) of any grade was reported in 6 patients (66.7%); however, no grade ≥ 3 CRS or any grade serious neurological events or treatment-related deaths were observed.

**Conclusion:**

Tisagenlecleucel showed high efficacy and manageable safety in adult Japanese patients with r/r FL. Moreover, the clinical outcomes were similar to the global population, which supports the potential of tisagenlecleucel in Japanese patients with r/r FL.

**Supplementary Information:**

The online version contains supplementary material available at 10.1007/s12185-022-03481-y.

## Introduction

Follicular lymphoma (FL), an indolent form of non-Hodgkin lymphoma (NHL), is considered incurable in most patients due to its typically relapsing-remitting pattern [1]. FL is the second most common subtype of NHL in Japan after diffuse large B-cell lymphoma (DLBCL) [2, 3]. The proportion of FL cases in Japan has increased from 6% of all lymphoid neoplasms within the period 1996‒2000 to 22.4% from 2007 to 2014, approaching rates observed in Western countries (28–31%) [3, 4]. Conventional therapeutic options for first-line therapy include watchful waiting, radiotherapy, and anti-CD20 monoclonal antibodies with or without chemotherapy [5]. Although chemo-immunotherapy has improved the survival outcomes (median overall survival [OS]: ~ 20 years) in patients with relapsed or refractory (r/r) FL, approximately 20% of patients with FL experience disease progression within 24 months (POD24) of initial chemo-immunotherapy with a median 5-year survival rate of 50%, and this subset of patients has a poor prognosis [6]. In addition, patients with r/r FL after the second line of therapy typically have worsening survival outcomes with each subsequent line of therapy [7]. Over the past decade, therapeutic approaches in Japan have evolved, with the approval of several novel agents for r/r FL (e.g., EZH2 inhibitor [tazemetostat] and lenalidomide plus rituximab combination) [7–9]. However, these agents have demonstrated modest efficacy and the need for long-term treatment, which contribute to sustained risk for severe toxicities and poor quality of life (QoL). As such, there remains an unmet need for effective therapy for patients with r/r FL who received ≥ 2 prior lines of therapy [7, 9].

Cellular therapies, specifically anti-CD19 chimeric antigen receptor T-cell (CAR-T) therapies, have demonstrated promising results in clinical trials. Based on the results from the ZUMA-5 trial, axicabtagene ciloleucel was approved by the US FDA for patients with r/r FL after ≥ 2 prior lines of therapy [10, 11]. Tisagenlecleucel, an autologous anti-CD19 CAR-T therapy, was approved for the treatment of pediatric and young adult patients with r/r B-cell acute lymphoblastic leukemia and for the treatment of adult patients with r/r DLBCL worldwide including Japan [12, 13], and recently for adult patients with r/r FL by both the US FDA and EMA [14, 15]. The global phase 2 ELARA trial demonstrated that tisagenlecleucel is effective in patients with r/r FL, including those who are at high-risk[16]. The primary endpoint, complete response rate (CRR), was achieved (69.1%) with an overall response rate (ORR) of 86.2% at a median follow-up of 16.6 months. Safety data were consistent with the established favorable safety profile of tisagenlecleucel as there were no cases of grade ≥ 3 cytokine release syndrome (CRS) and only one case (1%) of grade ≥ 3 immune effector cell-associated neurotoxicity syndrome within 8 weeks of infusion [16, 17]. Additionally, long-term follow-up data (median follow-up of > 5 years) from a pilot trial of tisagenlecleucel in 14 patients with r/r FL showed sustained and durable responses with ORR of 79% and 5-year progression-free survival (PFS) of 43%. The median duration of response (DOR) and overall survival (OS) was not reached with a 60% probability of being disease-free at 5 years [17].

Currently, no commercial CAR-T therapies are approved for indolent r/r FL in Japan and this highlights the need for clinical efficacy and safety data with CAR-T therapies in Japanese patients. Here, we report the results of the Japanese subset analysis from the ELARA trial investigating the efficacy, safety, and cellular kinetics of tisagenlecleucel in adult patients with r/r FL who received ≥ 2 prior lines of therapy.

## Methods

### Study design and patient population

**Fig. 1:**
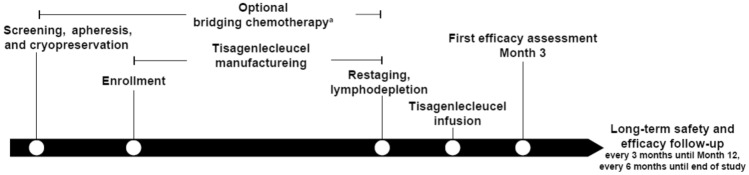
Study design. ^a^Disease was reassessed prior to infusion for all patients requiring bridging therapy

This Japanese subset analysis is based on patients included in ELARA (NCT03568461), a single-arm, multicenter, phase 2 trial of tisagenlecleucel in adults with r/r FL after ≥ 2 prior lines of therapy or who had relapsed after autologous stem cell transplantation (SCT). The global ELARA trial was conducted across 30 sites in 12 countries worldwide (including the United States, Europe, and Australia, and three sites in Japan) (Fig. 1) [16]. The key inclusion criteria were age ≥ 18 years, histologically confirmed by a central pathology review to have FL (grade 1, 2 or 3A), and r/r to ≥ 2 lines of prior therapy that included both an anti-CD20 antibody and an alkylating agent. The key exclusion criteria were patients who had evidence of histologic transformation, FL grade 3B, prior anti-CD19 therapy, prior adoptive T-cell therapy, prior gene therapy, prior allogeneic SCT or active central nervous system involvement by the malignancy.

Institutional approval from the Institutional Review Board/Independent Ethics Committee was obtained before initiating the trial. Written informed consent from all patients was obtained prior to screening and patients were enrolled in the study only when they met the clinical eligibility criteria, and their cryopreserved leukapheresis material was accepted by the manufacturing facility.

### Study treatments and administration

Tisagenlecleucel was manufactured in four sites across the world. A single intravenous infusion of tisagenlecleucel (protocol-specified dose ranging between 0.6 and 6 × 10^8^ CAR-positive viable T cells on day 1) was infused in an inpatient setting. Bridging therapy was given when needed at the investigator’s discretion, and the disease status was reassessed prior to tisagenlecleucel infusion to establish a new baseline in all patients. Lymphodepleting chemotherapy was given 1 week prior to tisagenlecleucel infusion. For lymphodepletion, patients could receive either fludarabine (25 mg/m^2^) and cyclophosphamide (250 mg/m^2^) daily for 3 days or bendamustine (90 mg/m^2^) daily for 2 days.

### Study endpoints

The primary endpoint was CRR based on the best response as determined by an independent review committee (IRC) using the Lugano 2014 classification [18]. Secondary endpoints included ORR, DOR, PFS, OS, safety, and cellular kinetics. Response assessments as per Lugano 2014 classification [18] were performed initially at 3 months after infusion followed by every 3 months during the first year after infusion and then every 6 months during the second year and through the end of the study (month 24). Patients with complete response (CR) or partial response (PR) were considered responders, while patients with progressive disease, stable disease, or unknown disease status were considered non-responders. Adverse events (AEs) were reported based on the Medical Dictionary for Regulatory Activities (MedDRA) version 24.0, and the Common Terminology Criteria for Adverse Events (CTCAE) version 4.03. CRS was graded using the Lee scale [19]. For cellular kinetics, tisagenlecleucel transgene levels in peripheral blood (transgene copies/µg of DNA) were measured by quantitative polymerase chain reaction (qPCR). Conventional pharmacokinetic parameters were calculated by non-compartmental methods (Phoenix WinNonlin, version 8.0) analyzing the assay results of qPCR and included the maximum level (*C*_max_), the time to reach the maximum level (*T*_max_), and the area under the transgene-time curve from day 0 to day 28 (AUC_0–28d_).

### Statistical analysis

This is a subgroup analysis of all patients enrolled in the ELARA trial from institutions located in Japan, with a data cutoff date (March 29, 2021) established after 90 infused patients in the overall study population were followed for at least 12 months from infusion or discontinued earlier. The efficacy analysis set (EAS) included all patients who received tisagenlecleucel infusion and had measurable disease pre-infusion per IRC review, the safety set included all infused patients, and the cellular kinetic analysis set included all patients in the EAS who provided at least one cellular kinetic parameter. All data analyses were descriptive owing to the small size of the Japanese subgroup. The CRR and ORR were reported with the 2-sided 95% exact Clopper-Pearson confidence interval (CI). The DOR, PFS, and OS were estimated using the Kaplan–Meier method, and their individual data were also presented along with the best overall response. The frequency and incidence proportion of AEs were reported, and cellular kinetics parameters were summarized using appropriate descriptive statistics. All data analyses were performed, and outputs were generated using SAS version 9.4. A detailed description of the overall study design and statistical analysis for the ELARA trial has been published previously [16].

## Results

### Baseline characteristics

As of March 29, 2021, nine patients were enrolled and infused with tisagenlecleucel across three study centers in Japan. All nine infused patients were included in the safety set, while eight patients were evaluable for efficacy due to no measurable disease in one patient at pre-infusion per IRC review (Figure S1). The demographics and baseline characteristics of all infused patients are summarized in Table 1. The median age of the patients was 61 years, and three patients were aged more than 65 years. Patients received three (range, 2–4) median prior lines of therapy and 66.7% (*n* = 6) of them received ≥ 3 prior lines of therapy. At study entry, 44.4% (*n* = 4/9) of patients had bulky disease, 77.8% (*n* = 7/9) had Ann Arbor stage III–IV disease, and 55.6% (*n* = 5/9) had a Follicular Lymphoma International Prognostic Index (FLIPI) score of ≥ 3. Additionally, 77.8% (n = 7/9) of patients had POD24 from first-line anti-CD20 monoclonal antibody-containing therapy, 44.4% (*n* = 4/9) had primary refractory disease, and 88.9% (*n* = 8/9) were refractory to the most recent line of therapy. All patients (9/9, 100%) received cyclophosphamide and fludarabine combination regimen as the lymphodepleting therapy. Bridging therapy was administered in 77.8% (*n* = 7/9) of patients and the most commonly used agents (≥ 20%) included rituximab, bendamustine (*n* = 3/9, 33.3% each), and etoposide, cyclophosphamide and dexamethasone (*n* = 2/9, 22.2% each).

**Table 1:** Demographic and disease characteristics (all infused patients)

Characteristics	Patients (*N* = 9)
Median age (range), year	61.0 (47‒71)
≥ 65 years, *n* (%)	3 (33.3)
Race: Asian, *n* (%)	9 (100)
Bone marrow involvement at study entry, *n* (%)	3 (33.3)
Histological grade at study entry: grade 1–2, *n* (%)	9 (100)
ECOG PS: 0/1/2, *n* (%)	5 (55.6)/3 (33.3)/1 (11.1)
Bulky disease at baseline^a^, *n* (%)	4 (44.4)
Ann arbor stage III‒IV at study entry, *n* (%)	7 (77.8)
Low/intermediate/high FLIPI at study entry, *n* (%)	1 (11.1)/3 (33.3)/5 (55.6)
Median no. of prior therapies (range)	3.0 (2‒4)
2/3/4 lines of therapy, *n* (%)	3 (33.3)/3 (33.3)/3 (33.3)
Prior autologous HSCT, *n* (%)	2 (22.2)
Prior therapy, *n* (%)	
Anti-CD20 mAb and alkylating agents^b^	9 (100)
Bendamustine based therapy^c^	9 (100)
PI3K inhibitors	0 (0.0)
Lenalidomide and rituximab	0 (0.0)
POD24 from first anti-CD20 mAb-containing therapy^d^, *n* (%)	7 (77.8)
Refractory to the last line of therapy^e^, *n* (%)	8 (88.9)
Primary refractory, *n* (%)	4 (44.4)
Refractory to ≥ 2 regimens^f^, *n* (%)	5 (55.6)
Double refractory^g^, *n* (%)	6 (66.7)

*CD* cluster of differentiation, *ECOG PS* eastern cooperative oncology group performance status, *FLIPI* follicular lymphoma international prognostic index, *HSCT* hematopoietic stem cell transplant, *mAb* monoclonal antibody, *PI3K *phosphatidylinositol 3-kinase, *POD24* progression of disease within 24 months

^a^Bulky disease was defined as one nodal or extranodal tumor mass > 7 cm in diameter or involvement of three or more nodal sites each of diameter > 3 cm

^b^Any regimen

^c^Median time from the last administration of bendamustine to the leukapheresis (*n* = 8; 1 unknown) was 23.46 months (range 1.2‒43.3), and the median number of absolute lymphocytes prior to leukapheresis in patients (*n* = 9) who received bendamustine was 0.830 × 10^9^/L (range: 0.37‒4.96)

^d^Disease progression < 24 months from initiation of first-line therapy, including primary refractory patients

^e^Refractory: failure to respond to previous treatment (stable or progressive disease as best response) or progression within 6 months of prior therapy completion

^f^Same or different regimens

^g^Patients who failed to respond or relapsed within 6 months following therapy with anti-CD20 and alkylating agents, any regimen

All patients received the recommended dose of tisagenlecleucel (0.6–6 × 10^8^ CAR-positive viable T cells) with a median infused dose of 2.35 × 10^8^ CAR-positive viable T cells (range 1.1–6.0 × 10^8^). The median time from enrolment to infusion was 63 days (range 51–120).

### Efficacy

**Fig. 2:**
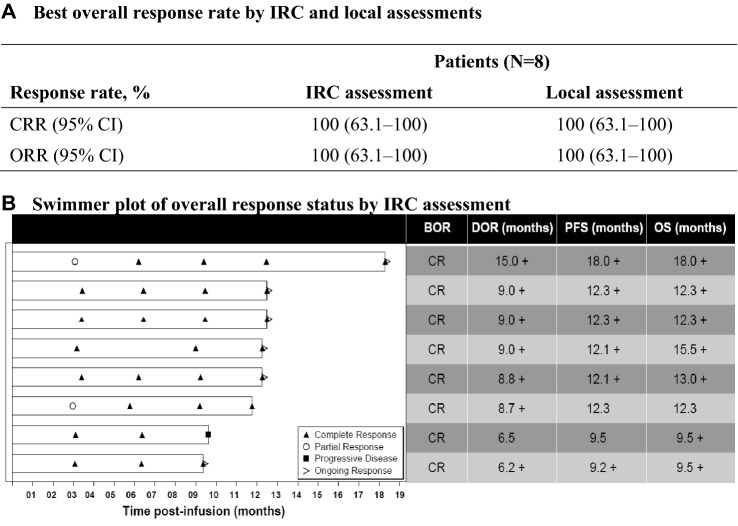
Response and survival outcomes (Efficacy analysis set). A patient who was excluded from the EAS due to no measurable disease at baseline per IRC achieved a BOR of PR with DOR 3.3 months, PFS 6.0 months and OS 12.0 + months. “ + ” denotes a censored observation. *BOR* best overall response, *CI* confidence interval, *CR* complete response, *CRR* complete response rate, *DOR* duration of response, *EAS* efficacy analysis set, *IRC* independent review committee, *ORR (CR + PR)* overall response rate, *OS* overall survival, *PFS* progression-free survival, *PR* partial response

**Fig. 3:**
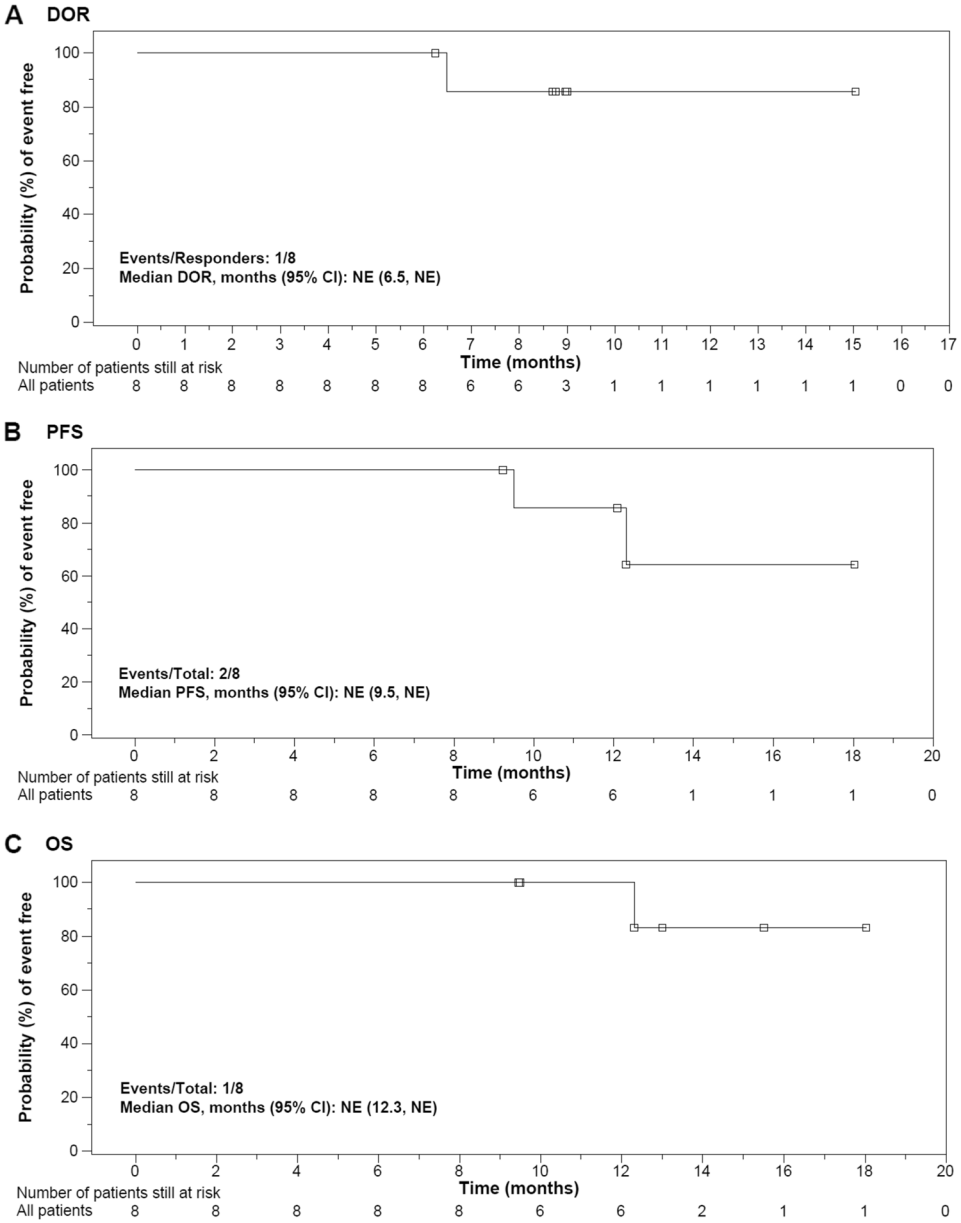
Kaplan–Meier curves of **A** DOR per IRC, **B** PFS per IRC and **C** OS (efficacy analysis set). **A** Time was relative to the onset of response. **B**, **C** Time was relative to tisagenlecleucel infusion. *CI* confidence interval, *DOR* duration of response, *IRC* independent review committee, *NE* not estimable, *OS* overall survival, *PFS* progression-free survival

At the data cutoff date, eight patients were evaluable for efficacy and 1 patient was not evaluable for efficacy due to no measurable disease pre-infusion per IRC review. Per IRC assessment, the CRR was 100% (8/8; 95% CI 63.1–100) in the EAS (Fig. 2). A high concordance was observed for the best overall response between IRC and local assessments. Median DOR, PFS, and OS were not reached (Fig. 3). After a median follow-up of 13.6 months (range 10.5‒19.3) post-tisagenlecleucel infusion, the estimated DOR rate at 9 months as per IRC was 85.7% (95% CI 33.4‒97.9), PFS rate at 12 months as per IRC was 85.7% (95% CI 33.4‒97.9), and OS rate at 12 months was 100% (95% CI 100‒100). Among all patients who achieved CR (*n* = 8) post- tisagenlecleucel infusion, one 47-year-old male patient who achieved CR at day 93 experienced disease relapse at day 289. This patient was presented with high-risk baseline disease characteristics such as stage IV disease, FLIPI score 4, bone marrow involvement, POD24 and bulky disease.

### Safety

Of the nine patients evaluable for safety, regardless of the treatment relationship, all patients experienced AEs of any grade and at least 1 grade ≥ 3 AE post-tisagenlecleucel infusion. The most common AEs of any grade included CRS and neutropenia (*n* = 6 each, 66.7%) followed by hypogammaglobulinemia (*n* = 5, 55.6%) and thrombocytopenia (*n* = 4, 44.4%). The most common grade ≥ 3 events were neutropenia (*n* = 6, 66.7%), febrile neutropenia, lymphopenia, and hypophosphatemia (*n* = 3, 33.3% each). At any time after the tisagenlecleucel infusion, treatment-related AEs of any grade were reported in all infused patients and grade ≥ 3 in 66.7% (*n* = 6) of patients. Within 8 weeks after infusion, 88.9% (*n* = 8) had at least 1 treatment-related AE; 55.6% (*n* = 5) had grade ≥ 3 events. Overall, one patient died > 30 days post-infusion; this event was initially attributed to CRS and later considered unrelated to tisagenlecleucel (Table 2). This patient was diagnosed on day seven with grade 1 CRS with no concurrent infection, which was later resolved. Subsequently on day 368, the patient experienced symptoms consistent with grade 2 CRS with concomitant organ toxicities, leading to death on day 375.

**Table 2:** Overall safety profile (safety analysis set)

Adverse events post-infusion *n* (%)	Patients (*N* = 9)
Any AEs (all grade)	9 (100)
Suspected to be treatment-related	9 (100)
Grade ≥ 3 AEs	9 (100)
Suspected to be treatment-related	6 (66.7)
Serious AEs	2 (22.2)
Suspected to be treatment-related	2 (22.2)
Deaths within 30 days post-infusion	0 (0.0)
Deaths > 30 days post-infusion	1 (11.1)^a^

*AE* adverse event

^a^This death was initially considered related to tisagenlecleucel by the investigator. Upon a detailed case review including autopsy data obtained after the data cutoff date, it was considered unrelated to tisagenlecleucel

Within the first 8 weeks after infusion, CRS events were observed in 66.7% of patients, all were either grade 1 or 2 per the Lee scale (Table 3). The median time to onset of CRS was 4 days (range 2–7). Among patients with CRS (*n* = 6), some received supportive care including intravenous fluids for hypotension (*n* = 1; 16.7%), oxygen supplementation for hypoxia (*n* = 1, 16.7%), total parenteral nutrition (*n* = 3, 50%), and systemic anti-cytokine therapy with tocilizumab and corticosteroids (*n* = 1, 16.7%); no patient with CRS was admitted to an intensive care unit. In patients with CRS, fever and concurrent infections were observed in 83.3% (*n* = 5) and 16.7% (*n* = 1) of patients, respectively (Table S1).

**Table 3:** Adverse events of special interest regardless of treatment relationship (safety analysis set)

AESI^a^ within 8 weeks post-infusion, *n* (%)	Patients (*N* = 9)	
	All grades	Grade ≥ 3
Cytokine release syndrome	6 (66.7)	0 (0.0)
Serious neurological events	0 (0.0)	0 (0.0)
Infections	1 (11.1)	1 (11.1)
Tumor lysis syndrome	0 (0.0)	0 (0.0)
Prolonged depletion of normal B cells or agammaglobulinemia	3 (33.3)	0 (0.0)
Hematological disorders including cytopenias^b^	8 (88.9)	8 (88.9)
Neutropenia	4 (44.4)	4 (44.4)
Thrombocytopenia	4 (44.4)	2 (22.2)
Lymphopenia	3 (33.3)	3 (33.3)
Febrile neutropenia	2 (22.2)	2 (22.2)

*AESI* adverse events of special interest

^a^AESIs were presented based on the important identified risks of tisagenlecleucel. ^b^Only hematological events reported in ≥ 2 patients were presented below

No serious neurological events were observed within the first 8 weeks after infusion (Table 3). Hematological disorders including cytopenias of any grade were observed in 88.9% (*n* = 8) of patients, all experienced grade ≥ 3 events within the first 8 weeks after infusion. The most commonly reported cytopenias of any grade included neutropenia 44.4% (*n* = 4), thrombocytopenia 44.4% (*n* = 4), lymphopenia 33.3% (*n* = 3), and febrile neutropenia 22.2% (n = 2). All experienced events were grade ≥ 3 except for thrombocytopenia in two patients; these were manageable and resolved with adequate treatment. There was one case of grade ≥ 3 infection (bacteremia) considered related to tisagenlecleucel that occurred within the first 8 weeks of infusion, which was resolved by antibiotics (cefepime, vancomycin, and levofloxacin). Prolonged depletion of normal B cells or agammaglobulinemia was observed in 55.6% (*n* = 5) of patients post-tisagenlecleucel infusion, all were grade 1/2 events and were ongoing at the data cutoff date. All these patients received either prophylactic or therapeutic intravenous immunoglobulins.

### Cellular kinetics

**Fig. 4:**
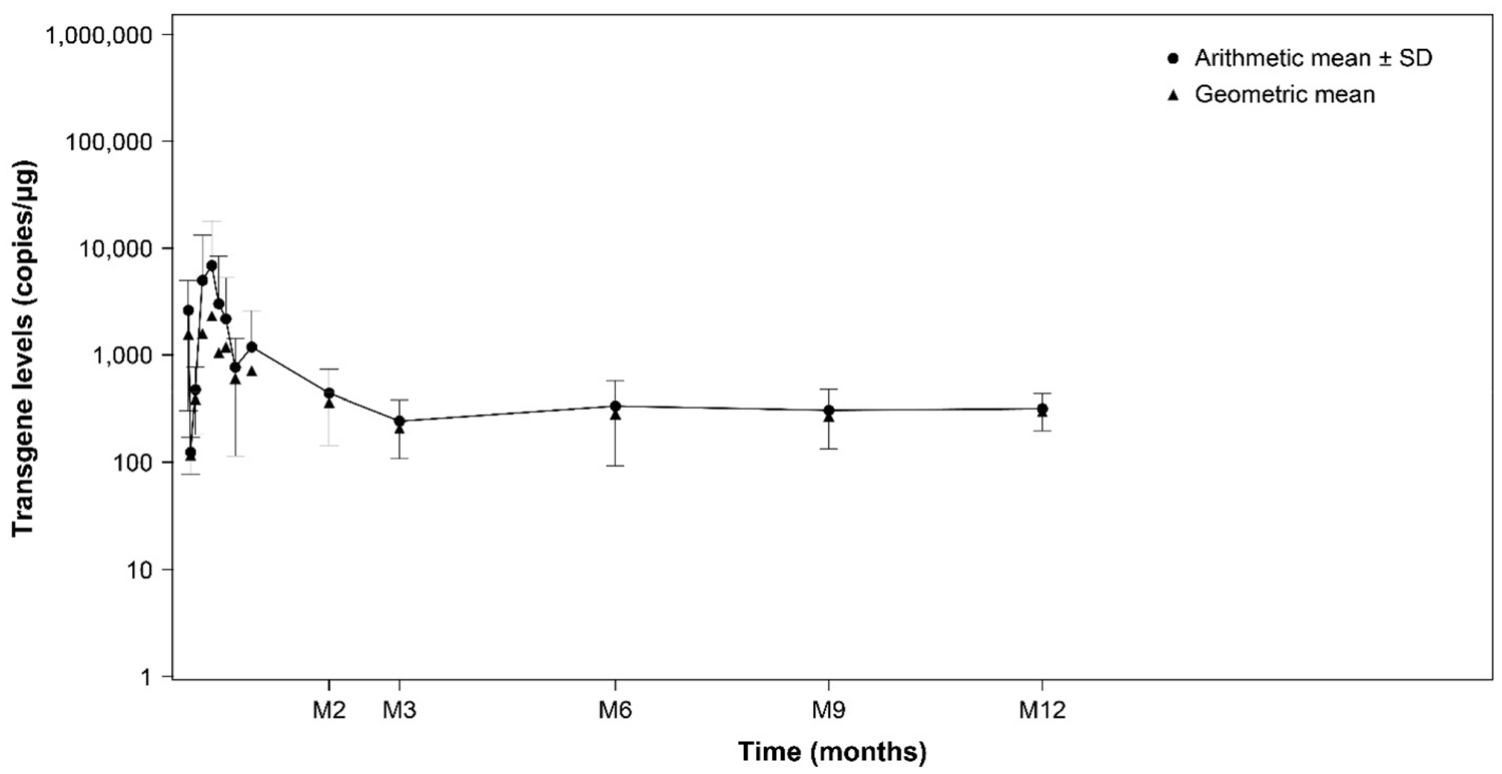
Time course of tisagenlecleucel transgene in peripheral blood (cellular kinetic analysis set). *Y-axis*: semi-logarithmic scale. *M in X-axis*: months, *n* = 8. *SD* standard deviation

Overall, eight patients were evaluable for cellular kinetic analysis. Tisagenlecleucel transgene in Japanese patients showed maximum expansion at day 9 with a lasting elimination phase (Fig. 4), which was similar to the result in the whole study population. The median (range) of *T*_max_, was 8.98 (6.8‒16.8) days (*n* = 7), median *C*_max_ was 2840 (548‒30,800) copies/μg (*n* = 7), and median AUC_0-28d_ was 34,700 (7940‒319,000) copies/μg∙days (*n* = 7). All Japanese patients had cellular kinetic parameters within the range observed in non-Japanese patients.

## Discussion

ELARA is a phase 2, multicenter, global, pivotal trial of tisagenlecleucel, a second-generation anti-CD19 CAR-T therapy [16]. This subgroup analysis of ELARA assessed the efficacy, safety, and cellular kinetic profile of tisagenlecleucel in adult Japanese patients with r/r FL. In Japan, standard-of-care treatment options available for patients with r/r FL after ≥ 2 prior lines of therapy are limited. Additionally, the clinical outcomes with the available treatments in this setting are poor and highlight the need for novel agents. [7]. To our knowledge, this is the first report of an anti-CD19 CAR-T therapy to address the poor prognosis in this patient setting. The results presented here demonstrate that tisagenlecleucel is an effective therapy with a manageable safety for adult Japanese patients with r/r FL who received ≥ 2 prior lines of therapy.

Tisagenlecleucel demonstrated high rates of durable response, with a CRR of 100% in Japanese patients compared with the overall study population (*N* = 94 in EAS; ORR, 86.2% and CRR, 69.1%) [16]. The numerically higher response rates might be because Japanese patients were treated at an earlier line of therapy with tisagenlecleucel than the overall study population. High response rates with durable responses, similar to tisagenlecleucel, have also been observed with other anti-CD19 CAR-T therapies, confirming the role of anti-CD19 CAR-T in the treatment of r/r FL [11]. The global ELARA trial confirmed the durable ongoing responses in patients with r/r FL, including in high-risk patients who achieved a response (CR or PR), suggesting possible sustained efficacy of tisagenlecleucel in the Japanese patients who achieved a response [16, 20].

Based on safety data reported in the global ELARA and ZUMA-5 trials, tisagenlecleucel (grade ≥ 3 CRS/neurological events: 0%/3% in the ELARA trial) could potentially be more tolerable than axicabtagene ciloleucel (grade ≥ 3 CRS/neurological events: 7%/19% in the ZUMA-5 trial), in this patient setting [11, 16, 21]. Safety data in Japanese patients are consistent with the established safety profile of tisagenlecleucel and are similar to the overall population [16]. Moreover, the safety profile of tisagenlecleucel in Japanese patients with r/r FL was similar to r/r patients with DLBCL observed in the JULIET trial [22]. No specific safety concerns were observed for Japanese patients. As of the data cutoff date, no grade ≥ 3 CRS or any grade serious neurological events were reported within 8 weeks post-infusion in Japanese patients. However, one grade 5 (fatal) event occurred in a 71-year-old male patient at 375 days post-infusion. Initially on day 7, this patient was diagnosed with grade 1 CRS per Lee scale with no concurrent infection, which was later resolved. Subsequently on day 368, the investigator reported the second episode of grade 2 CRS without fever–this was a diagnosis of exclusion, as work-up for both sepsis and autoimmune disorders was negative. The patient required treatment with vasopressin, tocilizumab (8 mg/kg, two doses) and high-dose corticosteroids (methylprednisolone 1 g) alongside low-flow oxygen. On day 374, the patient additionally received anti-human thymocyte IV immunoglobulin, adalimumab, and died on day 375. The cause of death initially was considered due to CRS (grade 5) per investigator assessment, with concomitant multiorgan toxicities of acute kidney injury, capillary leak syndrome and several other events such as stomatitis, pneumonia, sepsis, encephalopathy and upper gastrointestinal ulcer. Upon a detailed case review, including autopsy data obtained after the data cutoff date, the cause of death was considered to be multiorgan failure unrelated to tisagenlecleucel treatment. The autopsy results reported the diagnosis of macrophage activation syndrome (MAS) with no evidence of disease relapse. In addition, when CD19 staining was performed at the macrophage aggregation sites, including the brain, no CD19-expressing cells were observed and a causal relationship with tisagenlecleucel could not be identified. However, the report indicated that immunological abnormalities after tisagenlecleucel therapy caused MAS, which might have caused organ damage and massive effusion into a body cavity, prior to the eventual death of this patient with pneumonia and an aggravated systemic condition. The local pathologist also considered the newly reported MAS unrelated to tisagenlecleucel and the primary cause of death as multi-organ failure. In addition to the autopsy findings, the results of the CAR transgene assessment indicated that the transgene levels were already below the limit of quantification at 6 months and 3 months before death. Moreover, transgene levels were not detected from a bone marrow aspirate sample collected 2 weeks before death. No available test results supported an association with tisagenlecleucel.

Based on the transgene-time profiles and cellular kinetic parameters, cellular kinetics in Japanese patients were similar to those observed in the whole study population, suggesting ethnic insensitivity [16].

This report has some limitations to consider when interpreting the results. The analysis has a limited number of patients (*n* = 9) and a relatively short follow-up duration (median 13.6 months). Hence, to definitively corroborate the results and confirm the actual role of tisagenlecleucel in adult Japanese patients with r/r FL, further evaluation in a larger number of Japanese patients with a longer follow-up time would be required.

In summary, the results from the ELARA Japanese subset analysis of tisagenlecleucel showed high efficacy and a manageable safety profile in adult patients with r/r FL after ≥ 2 prior lines of therapy. The clinical outcomes in this subset analysis were promising (despite the limited number of patients) and are consistent with those of the overall ELARA study population. Tisagenlecleucel could offer a potential new treatment option for adult Japanese patients with r/r FL.

## Supplementary Information

Below is the link to the electronic supplementary material.

Supplementary file1 (DOCX 203 KB)

## Data Availability

Novartis is committed to sharing with qualified external researchers, access to patient-level data, and supporting clinical documents from eligible studies. These requests are reviewed and approved by an independent review panel on the basis of scientific merit. All data provided are anonymized to respect the privacy of patients who have participated in the trial in line with the applicable laws and regulations. The availability of trial data is according to the criteria and process described on https://www.clinicalstudydatarequest.com.
