# Supplementary material for: Efficacy and safety of tisagenlecleucel in adult Japanese patients with relapsed or refractory follicular lymphoma: results from the phase 2 ELARA trial
Source: Int J Hematol. 2022 Nov 21;117(2):251–9. doi: 10.1007/s12185-022-03481-y (PMC9889457; doi:10.1007/s12185-022-03481-y)
Supplement: Supplementary file 1 — Supplementary file1 (DOCX 203 KB) [file 12185_2022_3481_MOESM1_ESM.docx]

**SUPPLEMENTARY MATERIALS**

**TABLES**

**Table S1: Cytokine release syndrome (Safety analysis set)**

| **Characteristics of first CRS episode^a^, n (%)** | **Patients (N=9)** |
| --- | --- |
| Maximum CRS grade per Lee scale | 6 (66.7) |
| Grade 1 | 5 (55.6) |
| Grade 2 | 1 (11.1) |
| Among patients with CRS (n=6): |  |
| Fever | 5 (83.3) |
| Concurrent infections | 1 (16.7) |
| Median time to onset, days (range) | 4.0 (2‒7) |
| Median duration, days (range) | 9.5 (7‒24) |
| Admitted to ICU | 0 (0.0) |
| Patients with resolved events | 6 (100) |
| Management: |  |
| Total parenteral nutrition | 3 (50.0) |
| Tocilizumab | 1 (16.7) |
| Corticosteroids | 1 (16.7) |
| Intravenous fluids for hypotension | 1 (16.7) |
| Oxygen supplementation for hypoxia | 1 (16.7) |

^a^Only the first CRS episode post-infusion was summarized for each patient: all first episodes were reported within 8 weeks post-infusion. Only 1 patient had the second CRS episode reported >1 year post-infusion with maximum grade 5. CRS, cytokine release syndrome; ICU, intensive care unit.

**SUPPLEMENTARY FIGURES**

**Fig. S1: Disposition of the study patients (All enrolled patients)**


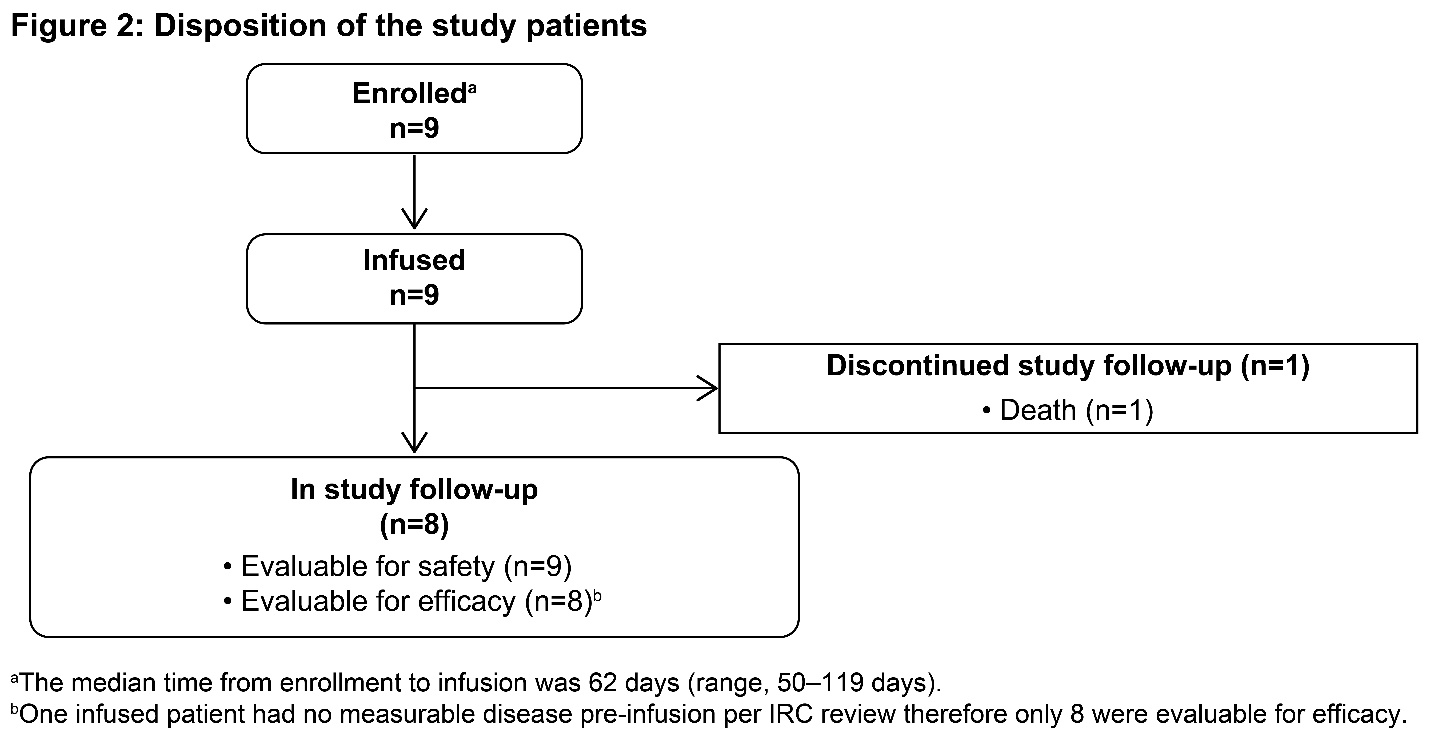


^a^The median time from enrollment to infusion was 63 days (range, 51–120). ^b^One infused patient had no measurable disease pre-infusion per IRC review; therefore, only 8 were evaluable for efficacy.
